# Anthropometric and Angular Measurements in Healthy Persian Females in Comparison to the Golden Ratio

**DOI:** 10.1111/jocd.16777

**Published:** 2025-01-16

**Authors:** Farideh Jokar, Maryam Hadibarhaghtalab, Hengameh Kasraei, S. Yasamin Parvar, Vahid Seifi, Zahra Vojdani Jahromi

**Affiliations:** ^1^ Department of Dermatology Shiraz University of Medical Sciences Shiraz Iran; ^2^ Molecular Dermatology Research Center, Dermatology Department Shiraz University of Medical Sciences Shiraz Iran; ^3^ Eye Research Center The Five Senses Health Institute, Rassoul Akram Hospital, Iran University of Medical Sciences Tehran Iran; ^4^ Student Research Committee Shiraz University of Medical Sciences Shiraz Iran; ^5^ Sports Medicine Research Center Neuroscience Institute, Tehran University of Medical Sciences Tehran Iran; ^6^ Department of Anatomical Sciences School of Medicine, Shiraz University of Medical Sciences Shiraz Iran

**Keywords:** Anthropometry, dermatology, facial dimensions, Persian, women

## Abstract

**Introduction:**

Anthropology is the scientific exploration of the human body morphology. The present study aims to establish the anthropometric norms among young Persian ethnic women and compare them with golden proportion, a mathematical formula in facial esthetics.

**Methods:**

This cross‐sectional study was performed on Persian women between July 2020 and January 2021. Persian women were randomly selected based on the inclusion criteria of the study. Two standard photos were taken of each participant in profile and frontal angle, and then the anthropometric ratios were extracted and compared in different groups.

**Results:**

Two hundred twenty‐five Persian women aged 20–50 years with an average age of 32.4 ± 7.09 were included. The golden ratio in Fars ethnicity was 1.79 ± 0.24. Forehead height I significantly increased with age (*p* value = 0.03). Philtrum length also showed a significant age‐related increase (*p* value = 0.001). Lower and upper lip heights increased with age (*p* value = 0.002). Our results revealed statistically significant differences in the mean labial fissure width among the three age groups (*p* value = 0.009). Lower vermilion height significantly decreased with increasing age (*p* value = 0.028). The mentolabial and nasal dorsum angles exhibited significant differences among the age groups (*p* value = 0.002). Furthermore, the jaw and chin angles were notably lower in the younger age groups (*p* value = 0.047 and 0.001, respectively). When comparing different ethnicities, the Turk ethnicity stood out as having a significantly higher chin angle.

**Conclusion:**

In conclusion, the present study challenges the universality of the golden ratio, with Persian females demonstrating a closer adherence to a ratio of 1.75 and recommending a modified golden ratio for Middle Eastern. Our findings also highlight the importance of considering age‐related changes in cosmetic interventions, particularly lip and forehead dimensions.

## 1 Introduction

The facial attractiveness of an individual stems from the harmonious coordination of its diverse components [1]. This appeal is markedly shaped by facial soft tissue, varying proportions, and factors such as gender, race, genetics, youthfulness, and symmetry [2]. Moreover, an individual's facial appearance significantly influences their social acceptance, mental well‐being, and self‐confidence [3].

Anthropology is the scientific exploration of the human body morphology and interpopulation differences and encompasses various factors contributing to the emergence of distinctions among populations, including genetics, environmental influences, and nutritional status [4]. Anthropometry, as a part of anthropology, focuses on measuring diverse dimensions in facial structure, seeking to delineate the ratios between different facial components, which exhibit variations among distinct racial groups [4]. Anthropometric ratios of the face are used in plastic and reconstructive surgery, orthodontic procedures, and treatment of genetic and acquired malformations as well as in art and forensic medicine [1]. Despite the prevalence of cosmetic and plastic surgeries in our region, there is a lack of studies specifically addressing anthropometric measurements. Furthermore, as these surgeries increasingly gravitate toward minimally invasive approaches, the availability of facial anthropometric proportions from native populations becomes pivotal, given their key role in these procedures [2].

In facial esthetics, there is a special mathematical formula called the golden ratio (GR). The GR or Phi number which is usually observed in nature shows a single point on a line that divides the line into two parts in such a way that the ratio of the smaller part to the larger part is similar to the ratio of the larger part to the whole line. This ratio is a repeating number that is rounded to 1.618 [5]. The eyes in the human face are located in the center of a Golden Rectangle made by the head. The nose and mouth are located apart based on the GRs between the eyes and the bottom edge of the chin [6]. In many orthodontics procedures, cosmetic procedures, rhinoplasty, plastic surgeries, and even forensic medicine, the GR can serve as a reference. It should not, however, be the only deciding tool when assessing face beauty [7]. The present study aims to establish the anthropometric norms among Persian ethnicity young women and compare them with golden proportion (GP). Due to the current world's rapid rate of international migration, it is essential for professionals such as dermatologists, plastic surgeons, and dental specialties to be aware of variations in normal facial characteristics among ethnic groups to refine cosmetic procedure outcomes, minimizing unnecessary interventions, and reducing complications [8].

## 2 Patients and Methods

This cross‐sectional study was performed on 20‐ to 50‐year‐old Persian women at a referral center (Shahid Faghihi Hospital, Shiraz, Iran) in the period between July 2020 and January 2021. Women who met the entry criteria were assigned to the study. The university ethics committee approved the study (Shiraz University of Medical Sciences, Shiraz, Iran [ethical code IR.SUMS.MED.REC.1399.207]) and complied with the Helsinki Declaration. Moreover, the participants were fully informed, the type of study was explained to them, and they provided signed consent preceding the study.

### 2.1 Participants

In this study, Persian women were randomly selected based on the inclusion criteria of not having any underlying diseases such as Cushing's syndrome, Bell's palsy, trigeminal neuralgia, sinusitis, stroke, scleroderma, depression, thyrotoxicosis, strabismus, bulimia nervosa, anorexia nervosa, and any other systemic, skeletal and mental disorders that have expressions on the face. In addition, they did not have a history of trauma and jaw and facial surgeries and plastic and cosmetic surgeries, pregnant and breastfeeding women, people who had filler injections, fat injections, and cosmetic procedures such as thread insertion in the last 2 years, martial arts, having dietary regimen history, and history of orthodontics that might affect the results of the study were not included in the study. In this research, the ethnicity of each person up to two previous generations (grandfathers and grandmothers) was considered. If the person was a hybrid, it was not included in the study. Because they constitute a small population of society, they do not threaten the external validity of the results.

### 2.2 Sample Size Measurement

The sample size was determined using MedCalc software version 22.019, power 80%, Type I error 5%, and information from the previous published article by Rahimi Jaberi et al. (*p* = 31.16 and effect size 0.1) in at least 183 people [4]. Therefore, a total number of 225 women entered the study.

$$n=\frac{{\left(Z_{1-\alpha /2}+Z_{1-\beta }\right)}^{2}\sigma^{2}}{d^{2}}.$$

We have compared three ethnicities of Fars, Turk, and Lor among the Persian women population. We have compared the results of 20 randomly chosen Fars participants with 20 Turk and 20 Lor groups.

### 2.3 Interventions and Outcomes

**FIGURE 1:**
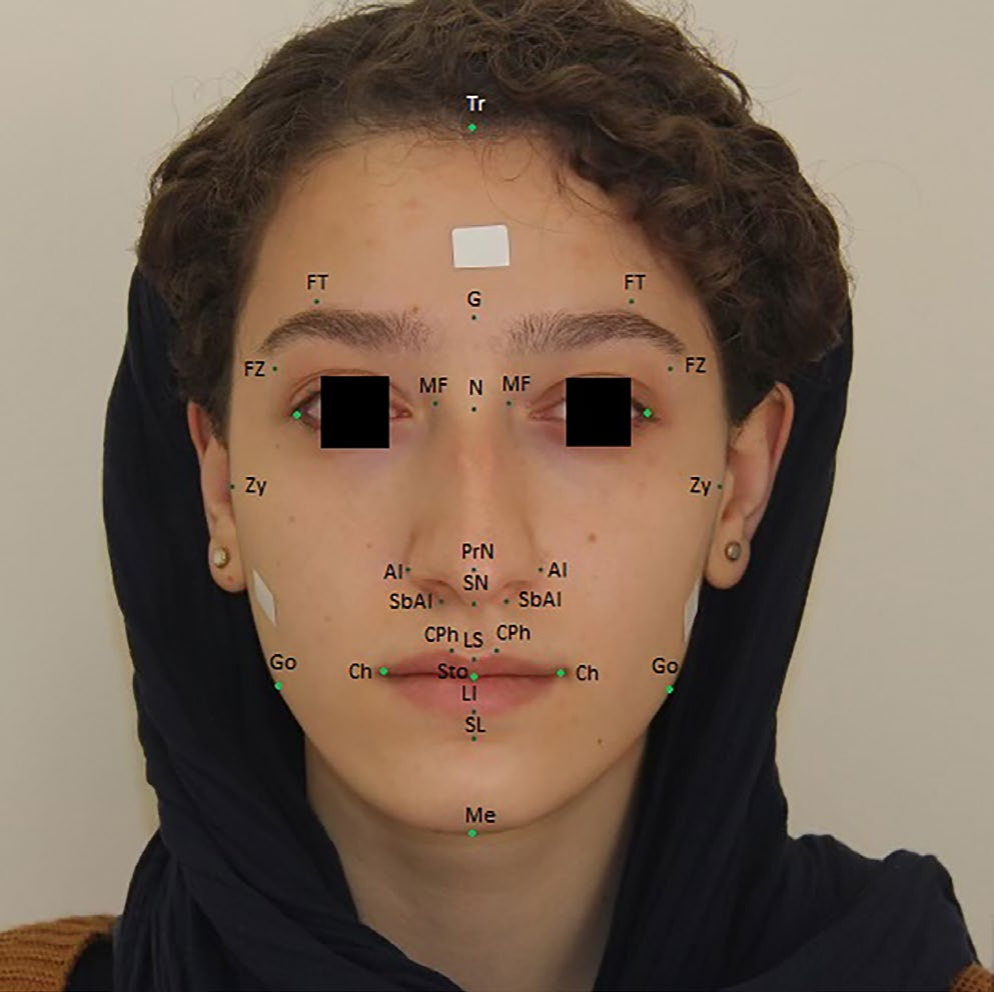
Facial landmarks in frontal angle. Al, alare; Ch, cheilion; CPh, crista philter; FT, frontotemporale; FZ, frontozygomaticus; G, glabella; Go, gonion; LC, lateral canthus; LI, labialeinferius; LS, labialesuperius; MF, maxillofrontale; Me, menton; N, nasion; PrN, pronasale; SbAl, subalare; SL, sublabiale; SN, subnasale; Sto, stomion; Tr, trichion; Zy, zygion.

**FIGURE 2:**
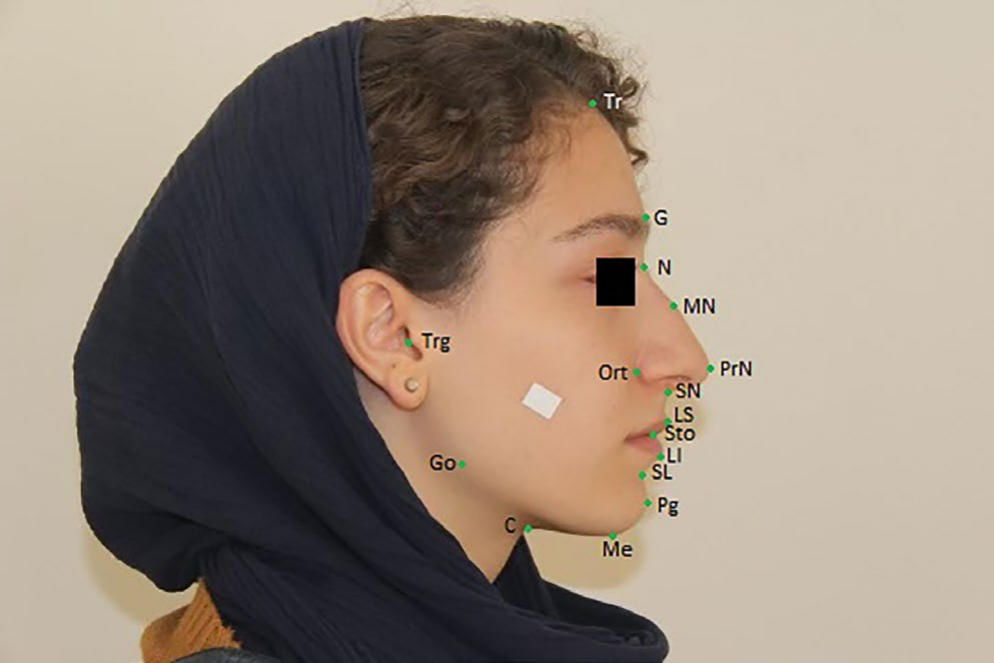
Facial landmarks in profile angle. C, cervical; G, glabella; LI, labialeinferius; LS, labialesuperius; Me, menton; MN, mid nasal; N, nasion; Ort point, junction of true vertical and true horizontal reference lines; Pg, pogonion; PrN, pronasale; SL, sublabiale; SN, subnasale; Tr, trichion; Trg, tragus.

Demographic information, including age, ethnicity, marital status, educational level, number of working hours per day, pregnancy history, and number of children, was obtained through a checklist. The dermatology resident took two photos of each person in this study in profile and frontal angle so that the line of hair growth up to the chin was clear. All photos were taken in a standardized condition with a white background and a Canon EOS 2000D professional camera. The camera stand was used to prevent shaking and sliding. The distance between the camera and the person was fixed at 2 m, and the visual axis was parallel to the floor. A measurement of 1.5 cm was taken in the middle of the forehead and in the cheek that was attached to the face to get the real size of the frontal and profile angles photos. People wearing glasses were asked to take off their glasses before taking pictures. Standardized face photographs were taken with eyes fully open, no smile, lips relaxed, teeth and jaw in a resting position, and forehead, neck, and ears defined. The participant's hair was off their face with a scarf. In all the photos used in this study, the people's eyes were covered by software, and their identities were not revealed (Figures 1 and 2). After collecting the required photos, the anthropometric ratios were extracted using the Adobe Photoshop CC 2020 v21.1.2.136 software, and the desired pixels were measured according to the standard tape; the actual sizes were determined, and the anthropometric scales of the face were presented by computer.

The anthropometric landmarks with abbreviations and measures used in this study and their definition are as follows:

- Tr (trichion): Midpoint of the hairline
- G (glabella): The most prominent point in the median sagittal plane between the supraorbital ridges
- N (nasion): The midpoint of the nasofrontal suture
- MN (mid‐nasal)
- MF (maxillofrontale): The anterior lacrimal crest of the maxilla at the frontomaxillary suture
- LC (lateral canthus)
- PrN (pronasale): The most protruded point of the nasal tip
- Al (alare): The most lateral point on the nasal ala
- SbAl (subalare): The point on the lower margin of the base of the nasal ala where the ala disappears in to the upper lip skin
- SN (subnasale): The junction between the lower border of the nasal septum, the partition that divides the nostrils, and the cutaneous portion of the upper lip in the midline
- CPh (crista philter): The point on the crest of the philtrum, the vertical groove in the median portion of the upper lip, just above the vermilion border
- LS (labialesuperius): The midpoint of the vermilion border of the upper lip
- Ch (cheilion): The outer corner of the mouth where the outer edges of the upper and lower vermilions meet
- Sto (stomion): The midpoint of the labial fissure when the lips are closed naturally
- LI (labialeinferius): The midpoint of the vermillion border of the lower lip
- SL (sublabiale): The midpoint of the labiomental sulcus
- Me (menton): The lowest point in the midline on the lower border of the chin
- Zy (zygion): The most lateral point on the zygomatic arch
- Go (gonion): The most lateral point at the angle of the mandible
- Pg (pogonion): The most forward‐projecting point on the anterior surface of the chin
- C (cervical): The most upper anterior cervical point in lateral position
- FT (frontotemporale): The most medial point on the temporal crest of the frontal bone
- FZ (frontozygomaticus): The most lateral point on the frontozygomatic suture
- Trg (tragus): A prominence on the inner side of the external ear

**Standard Anthropometric Measures**

- Ort point: Junction of true vertical and true horizontal reference lines
- Frontotemporale‐frontotemporale: Minimum frontal breadth/Horizontal line
- Frontozygomaticus‐frontozygomaticus: Supraorbital breadth/Horizontal line
- Trichion‐glabella: Forehead height I/Vertical line
- Trichion‐nasion: Forehead height II/Vertical line
- Glabella‐subnasale: Middle face height/Vertical line
- Zygion‐zygion: Maximum facial breadth/ Horizontal line
- Gonion‐gonion: Bigonial breadth/ Horizontal line
- Trichion‐Me(menton): Physiognomic face height/Vertical line
- Nasion‐gnathion: Morphologic face height/Vertical line
- Nasion‐stomion: Upper face height/Vertical line
- Stomion‐Me(menton): Anterior mandibular height/Vertical line
- Sublabiale‐gnathion: Chin height/Vertical line
- Subnasale‐gnathion: Lower face height/Vertical line
- Nasion‐subnasale: Nose height/Vertical line
- Nasion‐pronasale: Nasal bridge length/Vertical line
- Alare‐alare: Nose width/ Horizontal line
- Maxillofrontale‐maxillofrontale: Nasal root width/ Horizontal line
- Subalare‐subnasale: Nostril floor width/ Horizontal line
- Crista philtre‐crista philtre: Philtrum width/ Horizontal line
- Cheilion‐cheilion: Labial fissure width/ Horizontal line
- Subnasale‐labialesuperius: Philtrum length/Vertical line
- Labialesuperius‐stomion: Upper vermillion height/Vertical line
- Subnasale‐stomion: Upper lip height/Vertical line
- Stomion‐sublabiale: Lower lip height/Vertical line
- Labialeinferius‐sublabiale: Cutaneous lower lip height/Vertical line
- Labialeinferius‐stomion: Lower vermillion height/Vertical line

There are three ways to measure the GR. The first GR is determined by dividing the total length of the face by the width of the face; the second GR is the distance from the forehead to the bottom of the nose compared to the bottom of the nose to the chin, and the third GR is measured by the distance between the ends of the two eyes divided by the width of the lips.

### 2.4 Statistical Analysis

As the recent study is descriptive, standard deviation, mean, and frequencies were used. The data were tested for normality with Shapiro–Wilk test. Also, ANOVA was used to compare the average difference of parameters between age groups and face criteria, and if the data were nonparametric, its nonparametric equivalent, Cross‐Calvaris, was used. All calculations were done in IBM SPSS Statistics version 20 software. *p* value less than 0.05 was considered statistically significant for analyses.

## 3 Results

In this study, 225 Persian women aged 20–50 years with an average age of 32.4 ± 7.09 (mean ± standard deviation [SD]) were included. The demographic characteristics of the participants, such as marital status, history of pregnancy, educational level, and job, are shown in Table 1.

**TABLE 1:** Demographics and baseline characteristics of study participants (*n* = 225).

Age, years (mean ± SD)		32.4 ± 7.09
Marital status, *n* (%)		
	Single	100 (44.4)
	Married	125 (55.6)
History of pregnancy, *n* (%)		
	Positive	95 (42.2)
	Negative	130 (57.8)
Educational level, *n* (%)		
	Lower than diploma	8 (3.6)
	Diploma	62 (27.6)
	Bachelor	88 (39.1)
	Master	44 (19.6)
	PhD	23 (10.2)
Job, *n* (%)		
	Housewife	74 (32.9)
	Part‐time job	24 (10.7)
	Full‐time job	98 (43.6)
	Student	29 (12.9)

The first GR in Fars ethnicity was 1.79 ± 0.24. The second and third GRs were calculated as 1.66 ± 0.15 and 1.81 ± 0.18, respectively.

The general anthropometric size and different angles in Persian women are shown in Tables 2 and 3, respectively. These measurements are used to calculate the GRs.

**TABLE 2:** Anthropometric criteria and phi number by age and ethnicity.

Measurements	Group 1 20–29 years (mean ± SD in mm)	Group 2 30–39 years (mean ± SD in mm)	Group 3 40–50 years (mean ± SD in mm)	*p*	Fars ethnicity (mean ± SD in mm)	Turk ethnicity (mean ± SD in mm)	Lor ethnicity (mean ± SD in mm)	*p*
Minimum frontal breadth (FT_FT)	88.64 ± 7.48	86.09 ± 7.08	86.98 ± 7.40	0.055	87.19 ± 7.72	86.49 ± 9.06	88.41 ± 7.36	0.868
Supraorbital breadth (FZ_FZ)	103.13 ± 6.45	101.38 ± 6.60	101.59 ± 5.1	0.168	102.10 ± 6.40	101.28 ± 6.03	103.05 ± 7.53	***< 0.001***
Nasal root width (MF_MF)	22.46 ± 12.9	20.87 ± 2.29	21.21 ± 2.64	0.532	20.76 ± 8.70	20.38 ± 2.78	20.46 ± 2.02	0.209
Maximum facial breadth (Zy_Zy)	133.70 ± 13.95	135.47 ± 5.62	136.86 ± 5.67	0.561	134.16 ± 10.30	134.18 ± 4.50	135.17 ± 4.73	0.696
Nose width (Al_Al)	35.53 ± 2.60	36.16 ± 2.50	36.61 ± 2.33	0.48	36.05 ± 2.58	35.78 ± 2.32	35.89 ± 2.26	0.826
Nostril floor width (SbAl_SbAl)	20.53 ± 2.20	20.31 ± 2.55	20.95 ± 2.82	0.64	20.37 ± 2.40	19.92 ± 2.54	20.21 ± 3.33	0.255
Philtrum width (CPh_CPh)	11.17 ± 2.12	11.68 ± 2.16	11.30 ± 1.90	0.243	11.37 ± 2.12	11.06 ± 1.59	11.47 ± 2.77	0.755
Labial fissure width (Ch_Ch)	47.45 ± 3.57	48.96 ± 3.43	48.70 ± 3.05	***0.009***	48.35 ± 3.48	47.68 ± 4.19	48.16 ± 2.05	***0.021***
Bigonial breadth (Go_Go)	18.29 ± 70.7	109.13 ± 7.43	108.40 ± 9.76	0.755	107.99 ± 7.81	106.20 ± 7.84	108.62 ± 5.38	0.301
Forehead height I (Tr_G)	48.99 ± 7.14	51.3 ± 7.62	51.46 ± 6.43	***0.03***	51.36 ± 7.63	51.58 ± 4.46	50.96 ± 6.78	0.792
Forehead height II (Tr_N)	67.04 ± 6.97	68.97 ± 6.83	6 ± 6.74 ± 6.72	0.105	68.73 ± 7.91	69.61 ± 5.88	67.59 ± 7.72	0.573
Nasal bridge length (N‐PrN)	40.24 ± 3.53	40.27 ± 3.76	40.47 ± 3.78	0.996	40.20 ± 3.68	40.31 ± 4.00	40.11 ± 3.07	0.958
Philtrum length (SN_LS)	16.20 ± 2.05	16.60 ± 2.47	18.10 ± 1.95	***0.001***	16.56 ± 2.37	17.83 ± 1.88	16.91 ± 1.87	*0.03*
Upper vermilion height (LS_Sto)	15.87 ± 2.26	16.32 ± 3.59	16.19 ± 2.84	0.243	16.03 ± 2.73	16.37 ± 3.04	16.54 ± 3.19	0.568
Lower vermilion height (LI_sto)	9.54 ± 1.49	9.05 ± 1.58	8.78 ± 1.70	***0.028***	9.18 ± 1.61	9.14 ± 1.12	9.29 ± 1.98	0.136
Lower lip height (SN_Sto)	21.72 ± 2.36	22.02 ± 2.60	23.43 ± 2.45	***0.002***	21.99 ± 2.59	23.16 ± 2.31	22.64 ± 2.04	0.094
Physiognomical face height (Tr_Me)	181.95 ± 9.54	183.80 ± 9.86	180.42 ± 28.92	0.4	183.00 ± 15.48	186.81 ± 7.20	183.88 ± 8.51	0.119
Middle face height	67.50 ± 4.88	66.83 ± 4.84	65.90 ± 4.31	0.27	66.02 ± 4.88	68.66 ± 4.14	66.97 ± 3.81	0.240
Upper lip height (SN_Sto)	21.72 ± 2.36	22.02 ± 2.60	23.43 ± 2.45	***0.002***				0.094
Lower face height (SN_Me)	65.08 ± 4.56	65.63 ± 6.25	67.22 ± 4.41	0.310	65.29 ± 4.92	67.03 ± 4.10	69.17 ± 10.83	0.127
Nose height	50.19 ± 4.44	49.37 ± 3.30	49.87 ± 3.26	0.44	49.61 ± 3.42	51.13 ± 6.41	49.72 ± 2.34	0.624
Face height (Trg‐Ort)	96.20 ± 6.21	96.79 ± 6.79	96.79 ± 5.52	0.944	96.47 ± 6.41	96.05 ± 5.90	98.83 ± 6.42	0.421
Ort_PrN	29.61 ± 2.43	29.95 ± 2.71	29.84 ± 2.12	0.879	29.82 ± 2.54	29.52 ± 2.11	30.61 ± 2.69	0.374
N_Ort	44.69 ± 3.78	44.05 ± 3.55	44.02 ± 3.22	0.495	43.25 ± 3.68	44.32 ± 2.70	44.70 ± 3.71	0.694
C‐Me	33.84 ± 6.19	34.11 ± 6.29	33.29 ± 6.98	0.836	34.77 ± 6.49	32.93 ± 5.35	37.02 ± 5.11	0.101
G_Pg	120.67 ± 7.98	119.62 ± 8.67	120.91 ± 7.35	0.290	120.85 ± 8.43	121.57 ± 6.42	121.90 ± 7.40	0.327
Golden ratio 1	1.80	1.82	1.69		1.80	1.79	1.70	
Golden ratio 2	1.67	1.65	1.68		1.66	1.70	1.61	
Golden ratio 3	1.88	1.76	1.80		1.81	1.82	1.84	
Total mean of golden ratio	1.75							

*Note:* Bold italic values indicate statistically significant results with *p*‐values < 0.05.

**TABLE 3:** Facial angles across different age groups and ethnicities.

Measurments	Group 1 20–29 years (mean ± SD (degree))	Group 2 30–39 years (mean ± SD (degree))	Group 3 40–50 years (mean ± SD (degree))	*p*	Fars ethnicity (mean ± SD (degree))	Turk ethnicity (mean ± SD (degree))	Lor ethnicity (mean ± SD (degree))	*p*
G‐N‐PrN, nasofrontal	144.04 ± 10.74	144.87 ± 10.90	141.51 ± 8.26	0.686	148.27 ± 27.75	148.37 ± 7.75	142.77 ± 4.95	0.103
LI‐SL‐Pg, mentolabial	171.60 ± 6.98	171.78 ± 5.72	169.59 ± 9.34	***0.002***	170.98 ± 7.22	172.90 ± 5.05	174.15 ± 3.05	0.305
SN‐PrN/N‐PrN, nasal	172.50 ± 6.01	170.75 ± 12.37	172.73 ± 4.95	0.35	171.77 ± 9.85	171.81 ± 6.71	170.92 ± 6.85	0.287
N‐MN‐PrN, nasal dorsum	172.50 ± 6.01	170.75 ± 12.37	172.73 ± 4.95	***0.002***	94.32 ± 28.76	93.95 ± 7.24	93.15 ± 6.18	0.327
N‐Trg‐SN, mid facial	26.73 ± 2.32	26.22 ± 1.98	26.46 ± 1.43	0.145	35.88 ± 3.08	36.48 ± 2.48	34.92 ± 2.36	0.250
SN‐Trg‐Me inf facial third	36.22 ± 3.07	35.45 ± 3.07	36.35 ± 2.50	0.096	35.88 ± 2.36	36.48 ± 2.48	34.92 ± 2.36	0.295
G‐PrN‐Pg, total facial	136.94 ± 4.58	136.94 ± 4.85	137.86 ± 3.13	0.352	137.14 ± 4.56	136.19 ± 3.34	137.85 ± 5.32	0.560
Jaw angle	127.54 ± 6.72	130.10 ± 6.42	130.22 ± 6.96	***0.047***	128.79 ± 6.64	130.24 ± 8.41	128.92 ± 8.41	0.769
PrN_LS_SN, noselip	96.88 ± 9.55	94.81 ± 14.51	91.84 ± 11.09	0.0 52	94.88 ± 12.29	94.33 ± 12.05	99.15 ± 14.54	0.415
SL_Pg_Me, Chin angle	121.61 ± 11.68	125.75 ± 12.58	130.27 ± 12.90	***0.001***	124.61 ± 12.41	130.95 ± 12.78	120.54 ± 13.29	***0.037***
Trg/N					143.78 ± 10.16	147.14 ± 14.18	145.13 ± 12.15	0.440
PrN‐SN‐LS, nasolabial					143.78 ± 10.16	148.24 ± 7.71	142.23 ± 7.36	0.655

*Note:* Bold italic values indicate statistically significant results with *p*‐values < 0.05.

The comparison of anthropometric markers means in millimeters and three golden indexes among the age groups of 20–29, 30–39, and 40–50 years, and different ethnicities of Fars, Turks, and Lors was done separately using the ANOVA test.

In Table 2, we compared anthropometric criteria and the phi ratio across various age groups. Most anthropometric measures remained consistent with age. However, we observed that forehead height I (Tr_G) significantly increased with age (*p* value = 0.03), while philtrum length (SN_LS) also showed a significant age‐related increase (*p* value = 0.001). Both lower and upper lip heights increased with age (both *p* values = 0.002), suggesting a decrease in lip length and an associated increase in the distance between the nose and the lips.

Additionally, our results revealed statistically significant differences in the mean labial fissure width among the three age groups (*p* value = 0.009). Lower vermilion height (LI_Sto) significantly decreased with increasing age (*p* value = 0.028). Most anthropometric measures and phi ratios did not differ significantly across different ethnicities. However, we observed that supraorbital breadth (FZ_FZ), labial fissure width (Ch_Ch), and philtrum length (SN_LS) were significantly greater in the Lor, Fars, and Turk ethnicities, respectively (*p* values < 0.001, 0.021, and 0.03).

Table 3 presents variations in facial angles across different age groups and ethnicities. Notably, both the mentolabial angle and the nasal dorsum angle exhibited significant differences among the age groups (both *p* values = 0.002). Furthermore, the jaw and chin angles were notably lower in the younger age groups (*p* values = 0.047 and 0.001, respectively). When comparing different ethnicities, the Turk ethnicity stood out as having a significantly higher chin angle. Post hoc analysis revealed that Turkish people have a significantly different chin angle with Fars (PV = 0.037).

## 4 Discussion

With the shift from invasive esthetic surgeries toward minimally invasive procedures that necessitate a deep understanding of facial proportions specific to native populations, the scope of dermatologists and plastic surgeons has grown to reach optimal facial proportions to ensure ideal outcomes. Therefore, anthropometric analysis helps minimize unnecessary interventions and enhance patients' psychological health and self‐esteem [9]. The present study assessed anthropometric ratios of the face and their correlation with the GR and the difference among different age groups from 20 to 50 years participants and three ethnicities of Fars, Turk, and Lor. Most anthropometric measures and phi ratios did not differ significantly across different ethnicities. However, we observed that supra‐orbital breadth (FZ_FZ) was significantly greater in the Lor ethnicity, supraorbital breadth (FZ_FZ), and philtrum length (SN_LS) were significantly greater in the Fars and Turk ethnicities, respectively.

Many studies have been conducted on the GP in esthetic procedures. Segher et al. described the first use of the golden number in facial cosmetic surgery [10] and Rickets [11] is the first orthodontist who used the GR to assess the ideal proportions of a face. Interestingly, the closest average to the GR (Phi: 1.618) in our study was among the participants aged between 40 and 50 years (1.69) when dividing the total length of the face by the width of the face. The closest average to the second GR which was evaluated by the distance from the forehead to the bottom of the nose compared to the bottom of the nose to the chin was at the ages of 30–39 years (1.65). The results of our study show that the Fars ethnicity had the closest ratio to the phi number. The average total golden number among all ages and races in our study was reported to be 1.75. This ratio can be introduced for beauty in future studies of the Iranian population.

In the angle measurements across different ethnicities in our study, it was observed that only the chin angle was significantly greater in the Turkish ethnicity. Therefore, paying attention to this aspect is of great importance for beauty enhancement in Turkish women. In a study involving 133 Turkish cases aged 18–40, facial proportions were assessed using the phi index. The mean GR was 1.61 which was lower than our results (1.79) and very close to the phi index. Others tend to have a more elongated upper face relative to the lower face, emphasizing the importance of considering this in esthetic procedures for Turkish and neighborhood populations [12]. Another study was conducted in Japan on three groups including a group of normal Japanese women undergoing orthodontic treatment, a group of models, and actresses. Facial standards and the GR were examined, and it was determined that actresses have facial proportions of 100% corresponding to the golden number. While models had differences with the golden number due to having a short chin, and the normal women also had a big difference with the phi ratio and had a lower ratio and shorter faces than the actresses [13].

An ideal face's various horizontal and vertical dimensions are reported to adhere to the GR. Few writers have, nonetheless, produced contradictory findings. However, various populations throughout the world have varied ideas of what beauty is, and this could alter depending on the demographic group [7]. A recent study on the Turkish population revealed notable deviations in face width values and height from the GR [12, 14]. Furthermore, Rossetti et al. conducted a 3D stereophotogrammetry study on face esthetics, and GPs [15] found that the majority of facial ratios varied from the GR, making it impossible to establish a correlation between facial distance ratios and beauty [16]. Vietnamese women's soft‐tissue facial dimensions did not match the GP either [17].

According to the study of 150 participants and three ethnicities of Chinese, Indian, and Malay, only 22% of Chinese females had a facial ratio close to the golden number, 72% had a smaller proportion, and the rest were larger. In the Indian group, 20% had a facial ratio close to the golden number, and 78% were smaller. In the Malay group, only eight people (16%) had a ratio close to the GR, and the rest were smaller [18].

Heidari et al. performed a study on 18‐ to 25‐year‐old males from Baloch ethnicity in southern Iran highlighted that the lip index is larger in this ethnic group, and these men possess wider mouths [3]. Comparing anthropometric measurements across various age groups in the present study showed consistent trends except for the lower and upper lip heights which increased significantly with age, suggesting a decrease in lip length and an associated increase in the distance between the nose and the lips. Correcting these aspects can contribute to a more youthful appearance, particularly in the Iranian population. Therefore, attention to these factors in lip fillers and surgical interventions, such as lip centralization with lip lifts, can play a significant role in rejuvenating individuals.

In a study published in the *World Journal of Plastic Surgery*, involving 100 Iranian participants, facial proportions were compared with American. The findings revealed that the forehead length of Iranian women is significantly greater than that of American women. Additionally, the lower part of the face in Iranian women was observed to be taller compared to American women, aligning closely with the results of our study [19]. Similarly, we observed that the length of the forehead was significantly greater at higher ages. This could be attributed to the phenomenon of female hair loss due to hormonal and genetic factors. As a result, addressing and treating female hair loss at younger ages can significantly contribute to the beauty of women.

The nasal and labial LI–SL–Pg angle, specifically the mentolabial angle, was significantly greater in the younger age groups in our study. Xiong Jiang, and Liu show that injection of volume filler at the anterior nasal spine and the junction of philtrum and columella elevated the nasal base and increased the nasolabial angle. Therefore, by increasing this angle in older age groups through interventions such as Botox injections, fillers, and nasal surgery, facial esthetics can be directed toward a more youthful appearance [20].

In a survey conducted on 155 medical students with an average age of 22 years, 26 anthropometric parameters were compared between genders. Twenty‐two parameters exhibited significant differences between males and females, with the forehead and upper eyelid length being significantly greater in males. In the mid‐face region, no significant differences were observed, but in the lower face, males demonstrated both greater length and width [21]. Kaya and his collogues also evaluated 133 Turkish patients and seven studies around the world and concluded that facial height was significantly higher in males than females in almost all studies [12]. Distinct gender differences in facial angles were also observed in the Caucasian population [22]. Consequently, only females were included in our study to enable a more precise comparison of anthropometric averages.

To the best of our knowledge, this is the first anthropometric assay on Persian females evaluating the GRs in accordance with increasing age. Based on the outcomes of this study, a reevaluation of the GR is suggested for the Persian and Middle Eastern populations, which appears to be closer to 1.75. It is also recommended that further research with larger sample sizes be required to perform across diverse ethnicities to explore anthropometric differences geographically.

## 5 Conclusion

In conclusion, the present study challenges the universality of the GR, with Persian females demonstrating a closer adherence to a ratio of 1.75 and recommended a modified GR for Middle Eastern populations and encourages further research with larger and diverse samples to enhance the understanding of cultural and regional influences on facial esthetics. Our findings also highlight the importance of considering age‐related changes in cosmetic interventions, particularly in lip and forehead dimensions. The ideal proportions can not only save expenses and reduce patient dissatisfaction with cosmetic procedures but also can be used as a reference for planning orthodontic treatment, orthognathic surgical treatments like asymmetry correction, chin augmentation, and cosmetic surgeries like rhinoplasty and volume fillers even if it is not an absolute standard of facial attractiveness.

## Author Contributions

F.J. and M.H. conceived of the presented idea. M.H. and V.S. carried out the experiments. M.H., H.K., and S.Y.P. verified the analytical methods. F.J. and Z.V.J. supervised the findings of this work. All authors discussed the results and contributed to the final manuscript. S.Y.P. and H.K. took the lead in writing the manuscript. All authors provided critical feedback.

## Conflicts of Interest

The authors declare no conflicts of interest.

## Data Availability

The data that support the findings of this study are available upon request from the corresponding author. However, the authors are not publicly sharing the data due to privacy and ethical restrictions.
